# Alterations of gut microbiome in chronic rhinosinusitis: insights from a mendelian randomization study

**DOI:** 10.1016/j.bjorl.2025.101698

**Published:** 2025-09-20

**Authors:** Ke-Shuang Wang, Jun-Hao Tu, Qian-Xing Wang, Sui-Zi Zhou, Jia-Rong Wu, Qian-Hui Qiu

**Affiliations:** a Southern Medical University, Guangdong Provincial People's Hospital (Guangdong Academy of Medical Sciences), Department of Otolaryngology and Head and Neck Surgery, Guangzhou, China; b The First Affiliated Hospital of Nanchang University, Department of Otorhinolaryngology, Head and Neck Surgery, Nanchang, Jiangxi Province, China; c Affiliated Hospital of Guangdong Medical University, Department of Plastic Surgery, Guangdong Province, China

**Keywords:** Chronic rhinosinusitis, Gut microbiota, Mendelian randomization, Causality, Metabolic

## Abstract

**Objective:**

Gut microbiome dysbiosis is associated with various diseases. Causal association between Chronic Rhinosinusitis (CRS) and gut microbiome is yet unknown. This study aimed to investigate the potential causal relationship between CRS and gut microbiome dysbiosis.

**Methods:**

We used Genome-Wide Association Study (GWAS) data from FinnGen database for CRS. The Dutch Microbiome Project study provided data on gut microbiota species. A total of 334,182 individuals were included. Two-sample bidirectional Mendelian Randomization (MR) analysis was used to investigate causal relationship between CRS and gut microbiome. The main methods of evaluation were Inverse Variance Weighting (IVW), weighted median, weighted mode, and MR-Egger regression. Sensitivity analyses were performed to assess heterogeneity and pleiotropy.

**Results:**

Forward MR analysis indicated CRS is potentially linked to decreased risk of *Haemophilus parainfluenzae* (OR = 0.79, 95% CI 0.66‒0.94, *p* = 0.009) and increased risk of Bilophila’s (OR = 1.14, 95% CI 1.02–1.27, *p* = 0.023) within the gut. Reduced risks in gut microbiota-related pathways like UDP*-N-*acetyl-d-glucosamine biosynthesis I (OR = 0.85, 95% CI 0.77‒0.94, *p* = 0.002) and increased risk in pathway NAD biosynthesis I from aspartate (OR = 1.14, 95% CI 1.03–1.27, *p* = 0.010) were also linked to CRS. Reverse MR analyses, we obtained no positive results (*p* > 0.05/412).

**Conclusion:**

This study reveals CRS exerts a causal impact on shifts within the composition of the gut microbiome and also links to the changes of gut microbiota-related metabolic pathways. The risk of changes in gut microbiota should be of greater concern in patients with CRS than in the general population.

**Level of evidence:**

Mendelian Randomized (MR) studies are second only to randomized controlled trials in terms of the level of evidence.

## Highlights

• Chronic rhinosinusitis impacts shift in the gut microbiome composition.
• It may lower reduce *Haemophilus parainfluenzae* while increasing Bilophila.
• It may alter gut environment, causing a rise of the hazardous microbes Bilophila.
• It is linked to changes in gut microbiota's metabolic pathways.

## Introduction

Chronic Rhinosinusitis (CRS) represents a group of upper respiratory tract conditions caused by several immunopathological pathways that result in ongoing nose and sinus mucosal inflammation. CRS offers serious health problems for 5%‒12% of the general population, with main symptoms including nasal congestion, rhinorrhea, facial pain/pressure, and a diminished or lost sense of smell that lasts for more than 12 weeks.^1^ CRS is a crippling illness. It affects quality of life comparable to or greater than that of chronic bronchitis, asthma, peptic ulcer disease, Chronic Obstructive Pulmonary Disease (COPD), congestive heart failure, and angina.^1^, ^2^, ^3^

The human microbiome refers to the diverse group of bacteria that inhabit and interact with the human body, especially the immune system and epithelial cells on mucosal surfaces, such as airways.^4^ It plays a crucial role in digestion and protects against pathogenic microbial invasions. The EU Human Intestinal Metagenome Research Project team found that the genes contained in the human gut are 150 times more numerous than the human genome,^5^ labelled “the second genome of the human body”.^6^ In a large, multicenter international cohort of 410 CRS patients and healthy controls, Paramasivan et al. examined the nasal and sinus microbiota. They found that the genera Corynebacterium, Staphylococcus, Streptococcus, Moraxella, and Haemophilus made up the core microbiome in the nasal passages of both CRS patients and non-CRS individuals.^7^

The nasal cavity and the gut are anatomically linked and both have a diverse natural microbiota. Huang et al.^8^ used high-throughput sequencing techniques on faecal genomic DNA and discovered that the gut microbiota structure in the Chronic Rhinosinusitis with Nasal Polyps (CRSwNP) group differed significantly from the healthy control group. These changes were characterized mainly by an increased abundance of Saccharopolyspora and a decreased presence of Ruminococcaceae, Coprococcus, Collinsella, and Dialister. This study also predicted that these alterations are associated with changes in several metabolic pathways. Michalik et al.^9^ found that the indicative gut microbiota in CRS patients were altered, especially noting a decrease in Bifidobacterium, Lactobacillus mucosae, and Lactobacillus paracasei. This research suggests that measuring the levels of indicative gut bacteria could aid in planning more personalized and targeted probiotic treatments, potentially supporting the treatment of CRS patients.

Mendelian Randomization (MR)^10^ has found extensive use in genetic epidemiology. It has turned into a sophisticated and ideal genetic strategy that is increasingly being employed to identify the etiology of complicated diseases. Although there is literature associating CRS with gut microbiota dysbiosis, our current understanding of the relationship between them is limited and lacks clear causal evidence. This study uses the latest published data from Genome-Wide association Studies (GWAS) and employs two-sample MR analysis to systematically explore the causal relationship between CRS and the human gut microbiome. A deeper understanding of this causal relationship may provide important diagnostic and therapeutic approaches for CRS and gut microbiota dysbiosis.

## Methods

### Assumptions and study design

**Fig. 1:**
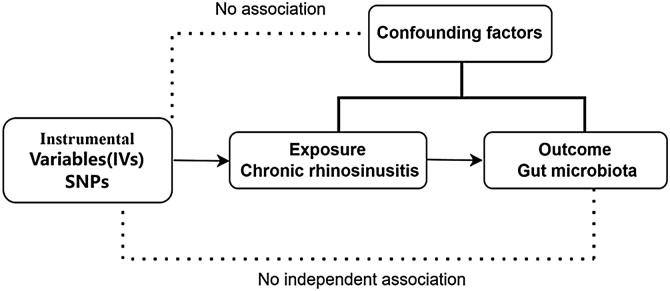
Mendelian randomization model of chronic rhinosinusitis and gut microbiota. *Note*: Confounding factors include antibiotic usage and aging.

As shown in Fig. 1, this work uses two-sample MR analysis to produce credible results. Meanwhile, this study is in line with the Strengthening the Reporting of Observational Studies in Epidemiology using Mendelian Randomization (STROBE-MR) guidelines.^11^ Randomized Controlled Trials (RCTs) are considered the gold standard in clinical research for examining causal relationships. But RCTs are often difficult to carry out. MR is an effective alternative that utilizes genetic variation as an Instrumental Variable (IV) to provide a robust method for detecting and quantifying causal relationships. Three essential criteria have to be met by IVs to ensure the validity of MR analysis:^12^ (1) Relevance: the genetic variation must be substantially linked to the exposure under research; (2) Independence: the genetic instrumental variable must be independent of any possible confounding influences; (3) Exclusion Limitation: the genetic instrumental variable should, by direct exposure, influence the result and not by other means.

**Fig. 2:**
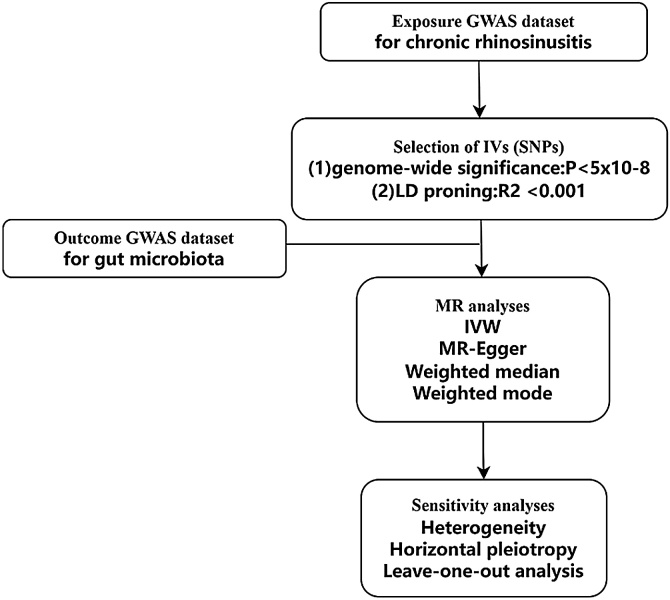
The schematic design and overview flowchart of this study.

The relevant analysis workflow is detailed in Fig. 2. This is the first MR study to analyze the impact of CRS on gut microbiota from a genetic perspective. Furthermore, the validation of horizontal multiple validity tests and association thresholds can further reduce erroneous causal inferences, thus increasing the credibility of the study results.

### Data sources

We used publicly available GWAS summary data in our MR analysis, as shown in Table 1. Conflicts of interest do not exist with these datasets. We utilized GWAS data from the latest FinnGen release on Chronic Rhinosinusitis (CRS) (finngen_R10_J10_CHRONSINUSITIS). 17,987 cases and 308,457 controls data were used to create the exposure dataset. Finland launched FinnGen in 2017, and it combines imputed genetic data from digital health registers and Finnish biobanks. The Netherlands Microbiome Project provided the information for 412 taxa of the gut microbiota. 207 microbial taxa and 205 pathways illustrating microbial composition and function were covered by GWAS in this study, which included 7738 people.^13^

**Table 1:** Details of the genome-wide association studies and datasets used in our analyses.

Items	Sample size (cases/controls)	Data sources	PMID	Data download link
CRS	17,987/308,457	FinnGen	NA	https://r10.finngen.fi/
412 Gut Microbiota	7738	DMP	35,115,690	https://www.ebi.ac.uk/gwas/; Accession numbersGCST90027446GCST90027857

CRS, Chronic Rhinosinusitis; DMP, The Dutch Microbiome Project; GWAS data is sourced from the most recently published database in 2024.

### Selection of SNPs

Using the three MR analysis assumptions, we chose Single Nucleotide Polymorphisms (SNPs) linked to CRS as IVs. Effective IVs were SNPs that showed a strong correlation with exposure (*p* < 5e-8). We employed Linkage Disequilibrium (LD)-based SNP clumping to identify independent instrumental variables. This process employed an *r*^2^ threshold of 0.001 and a clumping window of 10,000 kb, utilizing the LD reference panel from the 1000 Genomes Project. The SNP with the lowest p-value was retained.^14^ The F-statistic for the instrumental variables was calculated to assess the degree of weak instrument bias; an F-statistic greater than 10 was considered to indicate the absence of such bias. The effect size of the SNP is represented by beta, while the standard error of the effect size is denoted by se, in the formula F = beta^2^/se^2^.^15^^,^^16^ Following these guidelines is essential to guarantee the correctness and dependability of the outcomes of MR analysis as well as to help determine exact causal links between variables.

### Two-sample MR

Using the “TwoSampleMR” package in R software (version 4.3.1), we conducted bidirectional MR analysis to determine the causal relationship between CRS and 412 microbiological groups. Causal relationships were assessed using the Inverse Variance Weighted (IVW) model,^17^ the MR-Egger method,^18^ the Weighted Median,^19^ and the Weighted Mode.^19^ The IVW model is a weighted linear regression model and combines several IVs, each of which is weighted inversely proportional to its variance to reduce overall variance. The IVW approach served as the main foundation for the results, which were enhanced by additional approaches. Reverse MR analysis was carried out only when every MR technique confirmed the link between CRS and the gut microbiome. That is, the causal connection between gut microbiota exposure and CRS outcome was investigated. The research sample, procedure principle, and analysis processes for the reverse MR analysis were identical to those for the forward MR analysis. The direction of the causal effect was verified when the forward MR analysis was statistically significant, but the reverse MR analysis was not.

### Statistical analysis

We used MR-Egger regression to detect and correct for pleiotropy bias. We employed the Weighted Median method to guarantee accurate causal estimations, even in the case that up to 50% of the SNPs are invalid.^20^ For sensitivity analysis, we did the Cochran's *Q* test, MR-Egger regression and leave-one-out analysis. Accuracy and dependability of the MR analysis were increased when a p-value in Cochran's *Q* test was less than 0.05 and indicated statistically significant heterogeneity among the instrumental variables.^21^ Leave-one-out sensitivity analysis was used to track whether the presence of a single SNP driving substantial connections. The *R* program (version 4.3.1, accessible at http://www.r-project.org) was used to produce all statistical analyses and graphical results.

## Results

### Instrumental variable selection

We extracted a certain number of SNPs from the CRS dataset (finngen_R10_J10_CHRONSINUSITIS), all of which met our predetermined selection criteria. Specifically, the IVs significantly associated with CRS (*p* < 5 × 10^−8^) were identified, followed by LD pruning (r² < 0.001, 10,000-kb window). Each SNP had an F-statistic of at least 10, indicating a low chance of mild instrument bias. Supplementary Table 1 categorizes the included SNPs.

### Causal relationships between CRS and gut microbiota species

According to the IVW MR analysis results (Supplementary Table 2), CRS was genetically predicted to have a potential causal relationship with 12 gut microbiota species (*p* < 0.05). CRS was associated with an increased risk of 7 gut microbiota taxa and a decreased risk of 5 gut microbiota taxa. We found that CRS might impact gut microbiota, showing a potential causal relationship with decreased risks for the *Gammaproteobacteria*, *Pasteurellales*, *Pasteurellaceae*, *Haemophilus*, and *Haemophilus parainfluenzae*, all of which belong to the Proteobacteria. On the other hand, CRS was associated with an increased risk for the Deltaproteobacteria, Desulfovibrionales, Desulfovibrionaceae Bilophila, and unclassified Bilophila, which also belong to the Proteobacteria. Additionally, CRS showed a potential causal relationship with increased risks for *Alistipes indistinctus* in the *Alistipes.s* and *Odoribacter splanchnicus* in the *Odoribacter.s*, both of which belong to the Bacteroidetes.

**Fig. 3:**
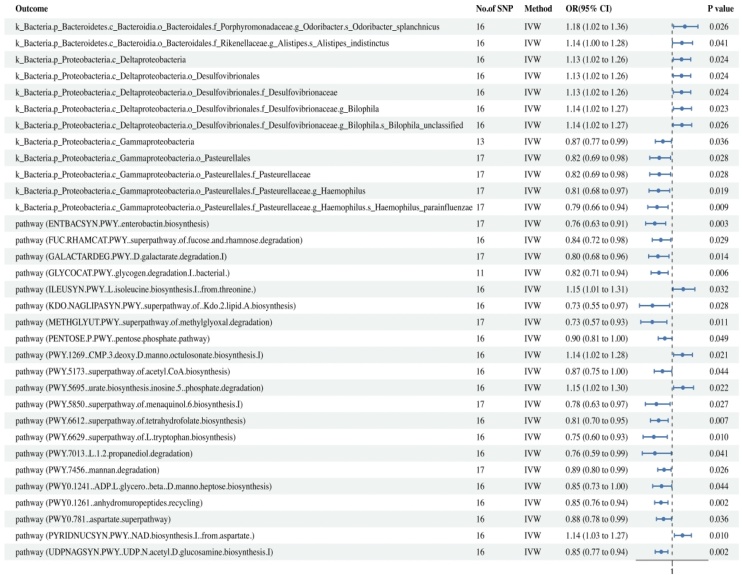
This forest plot displays the Mendelian Randomization assessment of the association between chronic rhinosinusitis and gut microbiota.

The results showed that CRS was most significantly associated with an increased risk of Bilophila (OR = 1.14, 95% CI 1.02–1.27, *p* = 0.023) and a decreased risk of *Haemophilus parainfluenzae* (OR = 0.79, 95% CI 0.66‒0.94, *p* = 0.009) within the gut. Consistent results were obtained through IVW, MR Egger, and weighted median causal association analyses (Fig. 3).

### Causal associations between CRS and gut microbiota metabolic pathways

The IVW MR analysis results (Supplementary Table 3) found CRS was genetically predicted to have causal relationships with 21 gut microbial functional metabolic pathways (*p* < 0.05). CRS was associated with an increased risk for 4 gut microbial-related metabolic pathways and a decreased risk for 17 gut microbial-related metabolic pathways. Among the pathways, CRS was most significantly associated with a decreased risk for the pathway (UDPNAGSYN.PWY..UDP.N-acetyl-d-glucosamine.biosynthesis.I) (OR = 0.85, 95% CI 0.77‒0.94, *p* = 0.002) and the pathway (PWY0-1261..anhydromuropeptides.recycling) (OR = 0.85, 95% CI 0.76‒0.94, *p* = 0.002), and an increased risk for the pathway (PYRIDNUCSYN.PWY..NAD.biosynthesis.I..from.aspartate) (OR = 1.14, 95% CI 1.03–1.27, *p* = 0.010). Results from IVW, weighted median, weighted mode, and MR Egger analyses are consistent, as shown in Supplementary Table 2 and Fig. 3.

### Sensitivity analyses

No pleiotropy was found by the MR-Egger regression intercept or the Cochran *Q* test (Supplementary Tables 2‒3). We also evaluated how each SNP affected the total causal estimate using leave-one-out analysis. The funnel and forest plots in this work showed no publication bias.

### Reverse MR analysis

When gut microbiota or signaling pathways were included as exposures, the genome-wide threshold for screening SNPs was set at 5 × 10^−10^. The reverse MR analysis revealed no significant causal relationship between gut microbiota/signaling pathways and CRS (*p* > 0.05/412) (Supplementary Table 4).

## Discussion

In this study, we revealed that CRS was associated with an increased risk for 7 gut microbiota taxa and a decreased risk for 5. CRS showed the most significant decreased risk association with *Haemophilus parainfluenzae* from the phylum Proteobacteria (OR = 0.79, 95% CI 0.66‒0.94, *p* = 0.009) and the most significant increased risk association with *Bilophila spp*. from the phylum Proteobacteria (OR = 1.14, 95% CI 1.02–1.27, *p* = 0.023). Similarly, CRS was linked to four increased and seventeen decreased gut microbiota-related metabolic pathways. CRS was most strongly associated with a decreased risk for the pathway (UDPNAGSYN.PWY.UDP.*N*-acetyl-d-glucosamine.biosynthesis.I) (OR = 0.85, 95% CI 0.77−0.94, *p* = 0.002) and the pathway (PWY0-1261.anhydromuropeptides.recycling) (OR = 0.85, 95% CI 0.76‒0.94, *p* = 0.002). Conversely, CRS was most notably associated with an increased risk for the pathway (PYRIDNUCSYN.PWY.NAD.biosynthesis.I.from.aspartate) (OR = 1.14, 95% CI 1.03–1.27, p = 0.010). Furthermore, the MR analyses performed in this study revealed no evidence of horizontal pleiotropy or heterogeneity, and sensitivity analysis confirmed the robustness of these findings.

The microbial genome of microbes that live on and within the human body, including the gastrointestinal system, urogenital tract, mouth cavity, nasal passages, and lungs, is referred to as the human microbiome.^22^ The interactions between the microbiota, as well as their interactions with the host, have a profound effect on the host immune system. The widespread presence indicates the potential microbe-related interactions between different organs.^23^ The gut microbiota is an intricate ecosystem of more than 100 trillion bacteria and is essential to maintaining host health.^24^ Numerous inflammatory and immune-related conditions, including metabolic, cardiovascular, allergy, and autoimmune disorders, are associated with an imbalance in the gut microbiota.^25^, ^26^, ^27^ The gut microbiota mainly consists of the phyla Firmicutes, Bacteroidetes, Actinobacteria, Proteobacteria, Akkermansia, and Fusobacteria.^5^^,^^28^^,^^29^

*Haemophilus parainfluenzae* (Hp) is a Gram-negative pleomorphic bacillus belonging to the phylum Proteobacteria. It lives mostly in the mucus of the nasal and oral canals and can cause various infections, including those of the ear, eye, sinus, and lungs. It is also an uncommon extraoral opportunistic pathogen.^30^ This bacterium is relatively often detected in the upper gastrointestinal tract of healthy individuals across various ages,^31^ as well as in inflammatory and faecal samples from children with diarrhea, though its prevalence is relatively low.^29^ The genus Bilophila, another member of the phylum Proteobacteria, is typically part of the normal colonic microbiota. It produces Hydrogen Sulfide (H_2_S), a genotoxic chemical that can harm intestinal epithelial cells. This damage has the potential to cause chronic intestinal disorders based on gene-environment interactions, as well as genomic instability or accumulating mutations in adenomatous polyps, eventually leading to colorectal cancer.^32^ Yang et al. further reported that H₂S-induced DNA damage may also contribute to chromosomal instability and cancer mutations, including those seen in small cell lung cancer.^33^

Previous studies have elucidated a significant increase of Proteobacteria in various chronic inflammatory disorders, including nonalcoholic fatty liver disease,^34^ Inflammatory Bowel Disease (IBD),^35^ asthma,^36^ and Chronic Obstructive Pulmonary Disease (COPD).^37^ Yibo Liang found that individuals with eosinophilic Chronic Rhinosinusitis with Nasal Polyps (eCRSwNPs) exhibited a markedly higher abundance of Proteobacteria at the phylum level compared to healthy controls.^38^ We also found the variations in the risk of Haemophilus and Bilophila within the Proteobacteria in CRS patients, indicating that CRS may modify the optimal intestinal environment for Proteobacteria. This change may cause a rise of the hazardous microbes Bilophila, increasing the risk of dysbiosis-related diseases such as colorectal cancer, small cell lung cancer, or tumor mutations.

CRS pathogenesis consists of damage to the epithelium barrier, compromised mucociliary clearance, and dysfunctional local host-environment interactions.^39^^,^^40^ Research studies reveal that immunological homeostasis, metabolism, and epithelial barrier function are all greatly impacted by the microbiota.^23^ Alterations in microbial composition are closely related to the disruption of the skin, gastrointestinal tract, and respiratory systems' mucosal barriers.^41^ The molecular foundation for changes in the gut microbiota of patients with CRS may include epithelial barrier disruption, inflammatory environment, and interactions with the host. How CRS-induced dysbiosis of the gut microbiota is caused by interactions between microbes and local immune responses. First, CRS is a chronic inflammatory disease of the nasal cavity and sinuses, which is marked by microbial infection and localized inflammation of the mucosa. This disturbs off the natural equilibrium of the microbial community.^42^ Second, the local immune disturbance may influence distal sites such as the gut, potentially altering gut microbiota composition. As indicated by the study, there may be a connection between the gut and nasal microbiota in CRS patients, but more research is required to understand the precise mechanism of this connection.^43^ Moreover, the common use of antibiotics to treat CRS patients may change the composition of gut microbiota, worsening the dysbiosis of the gut flora.^9^ Animal studies have also shown that changes in nasal microbiota and its metabolites can affect systemic health, demonstrating that dysbiosis may effect distant organs via microbial metabolites.^44^

The species of the gut microbiota with alterations brought about by CRS differ from those described in earlier research because of different data sources and experimental methods.^8^^,^^9^ According to this MR research, CRS is associated with increased risk of urate biosynthesis, NAD biosynthetic metabolic pathway of aspartate. Conversely, CRS is associated with reduced risks in the biosynthetic pathways for colicin, tetrahydrofolate, L-tryptophan, Kdo2-lipid A, ADP-L-glycero-β-d-manno-heptose, and UDP*-N-*acetyl-d-amino glucosamine. It also shows decreased degradation pathways for fucose, rhamnose, d-galactonate, bacterial glycogen, L-1,2-propanediol, mannan, methylglyoxal, the pentose phosphate pathway, the aspartate superpathway, and anhydrous peptide recycling. The observed changes in microbial species and metabolic pathways indicate that CRS may affect the gut microbiota's biological balance by decreasing metabolic activity. However, these changes in metabolic pathway levels are predicated on projections from sequencing data, and additional experimental confirmation is required to determine the actual metabolite level changes. From the perspective of MR, our findings highlight a disturbed gut microbiota in CRS patients, providing new insights into the disease's diverse pathogenesis and laying the groundwork for future intestinal metagenomic research in this population.

Our findings suggest that targeting the gut microbiota may offer novel therapeutic strategies for CRS treatment options. Some bacterial particles, vaccinations, bacterial lysates (dead cells), and live probiotic strains have been shown in studies to have immunostimulant characteristics.^9^^,^^45^ Clinical evidence has demonstrated significantly fewer infections and lower antibiotic reliance among children receiving bacterial lysates compared to placebo controls.^46^^,^^47^ Enhancing gut microbiota composition and barrier function may help prevent pathogenic invasion by modulating host immune responses. As a result, microbiota-based therapy, including as probiotic supplements and microbiome-targeted medicines, provide intriguing alternatives to traditional CRS treatments.

In previously published observational studies, the small sample sizes, low levels of evidence, and inconsistencies at baseline lead to questionable reliability of results. Excluding the effect of confounding factors (antibiotic usage and aging), our study investigated the causal relationship between CRS and gut microbiome and drew more reliable conclusions. This study also has some limitations. First, the population included in this study was European, and the lack of data on other ethnicities may have implications for our causality. The genetic makeup of various populations may produce differing outcomes that call for additional research. Second, our investigation only utilized datasets related to CRS without the phenotypes or endotypes of CRS. This issue may influence the outcomes, especially considering the pathophysiological differences among CRS endotypes. Our understanding of the relationship between CRS and gut flora would be enhanced by additional studies exploring the effects of different endotypes of CRS. Finally, even with the use of SNPs as a proxy for reducing potential bias, the underlying processes of the causal relationships shown in the study need to be further confirmed in well-designed investigations, such as experimental mechanistic research models.

## Conclusions

In conclusion, we found the variations in the risk of Haemophilus and Bilophila within the Proteobacteria in CRS patients, indicating that CRS may modify the optimal intestinal environment for Proteobacteria. Our study showed that CRS increases the risk of gut microbiome dysbiosis, potentially serving as a risk factor for alterations in gut microbiome composition and disruptions in gut microbiota-related metabolic pathways. This finding provides additional support for further investigation into the mechanisms underlying the crosstalk between CRS and the gut microbiome.

## ORCID IDs

Ke-Shuang Wang: 0009-0001-8219-6988

Jun-Hao Tu: 0000-0002-1306-0323

Qian-Xing Wang: 0009-0007-9584-9566

Sui-Zi Zhou: 0000-0002-8457-6044

Jia-Rong Wu: 0009-0000-7276-9140

Qian-Hui Qiu: 0000-0001-6335-5173

## Funding

This study was supported by the 10.13039/501100001809National Natural Science Foundation of China nº 82171104 (to Qian-Hui Qiu), the 10.13039/501100004543China Scholarship Council (nº 202308440505).

## Declaration of competing interest

The authors declare no conflicts of interest.
